# Critical appraisal of progress and challenges in tuberculosis preventive treatment in the Western Pacific Region: a situational analysis of seven high tuberculosis burden countries

**DOI:** 10.1186/s41182-025-00840-3

**Published:** 2025-10-27

**Authors:** Muhamad Khizar, Ehsanullah Alokozay, Muhammad Junaid, Najibullah Alokozay

**Affiliations:** 1 Manifest Medical Research Co., Georgian American University, Tbilisi, Georgia; 2 Faculty of Medicine, Kandahar University, Kandahar, Afghanistan; 3 Lady Readers Hospital, Peshawar, Pakistan; 4 Kabul University of Medical Science “Abu Ali Sina”, Kabul, Afghanistan

## Abstract

We commend **Oh et al.’s** recent analysis of TB preventive treatment (TPT) in the Western Pacific Region, but note important gaps and ways forward. We first caution that reliance on routine program data may overestimate gains. For example, China’s passive surveillance misses ≈20% of cases [1]. Prospective cohorts or integrated surveillance including clinical databases could validate coverage estimates. We also urge attention to overlooked risk groups beyond children and PLHIV (as highlighted by Oh et al. [2]), groups like healthcare workers, prisoners, and people with diabetes warrant targeted TPT pilots (e.g., occupational health or prison-based programs). In the Philippines, ~ 36% of TB patients first seek private care [3], so partnering with private clinics and pharmacies is essential to reach all contacts. Likewise, MDR-TB contacts were underemphasized; WHO now strongly recommends a 6-month levofloxacin regimen for MDR contacts [4]. We encourage pilot studies of this regimen (as in Mongolia [5]) and operational research on MDR-TPT. Finally, policy does not guarantee practice as Cambodia and Lao PDR have guidelines, yet stockouts and training gaps persist [6, 7]. Embedding TPT in universal health insurance and conducting cost effectiveness studies will support sustainable scale-up. In sum, by suggesting concrete examples and research strategies for each country, we aim to refine Oh et al.’s insights into actionable steps for TPT acceleration.

## To the editor:

### Dear editor,

We read with interest **Oh et al.’s** situational analysis of TPT in seven Western Pacific countries [1]. This valuable work identifies progress (e.g., adoption of shorter regimens in five countries [1]) and highlights age group disparities. We offer suggestions to strengthen the analysis and inform future action.

Oh et al. draw largely on program reports and expert consultations [1]. However, routine data may not capture the full picture. For instance, China’s TB Program estimates **≈95% of cases are detected by passive surveillance, implying ~ 20% remain undiagnosed** [2]. Similar under detection likely affects countries with remote areas (e.g., Lao PDR) or limited diagnostics. We suggest supplementing program data with prospective cohort studies or linked clinical-surveillance databases. For example, integrating TPT registries with national electronic health records could validate whether all identified contacts initiate therapy.

Oh et al. rightly note gaps in child and HIV-positive contacts [1], but other high-risk groups also merit focus. **Healthcare workers, prisoners, migrants and people with diabetes face elevated TB risk. WHO recommends TPT for many of these groups** [3], yet policies vary.

Notably, Mongolia’s guidelines include all WHO designated risk groups, whereas Lao PDR and PNG include none [3]. Pilot studies could explore targeted interventions: e.g., a cohort study of healthcare workers in Viet Nam or a pilot TPT program in PNG prisons. In Pacific islands (high TB/diabetes burden), screening diabetic clinics could identify latent TB. Addressing these neglected populations is essential to truly “reach” all who need TPT.

A key omission is the private health sector. In the Philippines, an estimated 36% of TB patients first seek care privately [4], yet private providers rarely report contacts or TPT uptake. Cambodia and Vietnam likewise have vibrant private clinics and pharmacies. Engaging these providers through mandatory reporting, public private partnerships or incentive models could substantially increase contact screening. For instance, pilots in Metro Manila have shown the feasibility of franchised TB care. Future research should evaluate how to integrate private-sector data into national TPT programs and fund private sector TPT delivery.

Preventive therapy for MDR-TB contacts also deserves more attention. **Recent WHO guidelines now strongly recommend daily levofloxacin for 6 months for MDR exposures** [5]. Mongolia is one of few countries adopting this regimen [6]. Other countries (e.g., Viet Nam, Philippines) have rising MDR-TB but lack data on MDR-TPT. We urge feasibility studies or implementation trials of 6Lfx in household contacts of MDR cases, including monitoring for adverse events. These could be small, pragmatic studies to build local evidence on MDR-TPT outcomes.

Oh et al. note that six of seven countries have TPT guidelines [1], but existence of policy does not ensure coverage. **In Cambodia, program reports highlight that LTBI treatment outcomes are often unreported outside HIV clinics** [7]. In Lao PDR, pediatric formulations are scarce and supply chains sporadic (only 10% of child contacts received TPT [8]). We recommend operational research to map policy practice gaps.

For example, supply chain audits to document INH/rifapentine stockouts, and staff surveys on TPT training. Strengthening data systems (e.g., merging TB and HIV registries) can track attrition at each cascade step, as suggested in recent discussions [7].

Finally, **sustainable financing is critical. Countries vary widely: Mongolia reports 63% domestic TB funding **[9]**, whereas Vietnam reports only 4% domestic funding with 75% of its TB budget unfunded** [10]. Cambodia and Lao PDR similarly rely heavily on donors. As countries transition from Global Fund grants, economic analyses are needed. Cost effectiveness studies of shorter regimens (as done in the Philippines) or modeling the impact of insuring TPT costs can guide funding decisions. Integrating TPT into national health insurance or community financing schemes would bolster sustainability.

In summary, Oh et al. provide an important regional overview of TPT progress. Building on their work, we suggest complementary approaches:

- validate coverage with cohort data, include all high-risk groups, partner with private providers, pilot MDR regimens, and assess health system constraints. Concrete examples from each country **(e.g., Cambodia’s low pediatric coverage **[7]***, the Philippines’ private-sector caseload ***[4]***, PNG’s moderate child-TPT uptake ***[11]***, Mongolia’s comprehensive policy ***[3]**)** illustrate that context-specific strategies are needed. Together, these measures can help ensure TPT translates from policy into practice across the Western Pacific.

## Data Availability

My manuscript has no associated data.

## References

[1] Oh KH, Teo AKJ, Yanagawa M, et al. Progress and challenges in tuberculosis preventive treatment in the Western Pacific Region. Trop Med Health. 2025;53:122. 40890887 10.1186/s41182-025-00805-6 PMC12403340

[2] China CDC Weekly. TB Surveillance Report. 2025.

[3] WHO–WPRO. Mongolia TB financing profile. 2024.

[4] Sherpa S, et al. TB patient pathways in the Philippines. Int J Tuberc Lung Dis. 2019;101:498.

[5] WHO. Latent TB Infection: updated and Consolidated Guidelines. 2024. 30277688

[6] WHO Global TB Report (2024): Mongolia country profile.

[7] Cambodia National TB Program Annual Report. 2023.

[8] WHO–WPRO. Lao PDR TB profile. 2024.

[9] WHO–WPRO. Vietnam TB financing profile. 2024.

[10] WHO–WPRO. PNG TB program update. 2024.

[11] Manoharan E. Facilitators and barriers for tuberculosis preventive treatment among patients with latent tuberculosis infection: a qualitative study. BMC Infect Dis. 2023;23(1):624. 37740196 10.1186/s12879-023-08612-2 PMC10517541

